# Role of Parental Supervision on Digital Screen Use and Its Effects on Children's Mental Health and Wellbeing in Bangladesh: A Cross Sectional Study

**DOI:** 10.1192/bjo.2024.179

**Published:** 2024-08-01

**Authors:** Hafiz Shahria Kakon, Rashid Tanjir Soron, Mohammad Shorif Hossain, Rashidul Haque, Fahmida Tofail

**Affiliations:** 1 International Center for Diarrheal Disease Research Bangladesh, Dhaka, Bangladesh; 2 Tele psychiatry Research and Innovation Network Ltd, Dhaka, Bangladesh

## Abstract

**Aims:**

The younger generation of today is highly dependent on digital technologies worldwide. Studies on young children's cognitive and socio-emotional development have shown that there can be conflicting effects from using screen-based media or from exposing them to it. The study explored the relation between unsupervised use of digital screen time with student mental health and behavioral problems.

**Methods:**

It was a cross sectional descriptive study approached primary and secondary school going children from grades 2–8 (age 6 to 14 years), purposively selected six schools consist of three English and Bangla medium schools from Dhaka city. A total of 420 students along with their parents were enrolled by clustered random sampling. Study explored the effect of the unsupervised screen time on student mental health and social wellbeing through semi structured questionnaires, Strength and Difficulties Questionnaire (SDQ), Pittsburgh Quality of Sleep Scale (PSQI), Spencer Children Anxiety Scale (SCAS) and Development and Wellbeing Assessment Scale (DAWBA).

**Results:**

Students used various forms of digital screens for 4.6 hours every day, and 56% of them used these devices without parental supervision or monitoring. English Medium students spend significantly more time on screens on a daily average (5.5 hours) compared with students at Bangla Medium schools (3.7 hours). 21.2% students had mental health concern, this percentage was higher in the unsupervised group (56.2%) than in the supervised group (43.8%). In the unsupervised group, students experienced higher levels of emotional difficulties (15.7%), behavioral difficulties (28.3%), hyperactivity behavior difficulties (17.4%), peer relations difficulties (28.8%), and pro-social behavior difficulties (6.7%) compared with supervised group. 83.3% of students in the supervised group found higher levels of anxiety compared with the unsupervised group (16.7). In the unsupervised group, 15.4% of the students had experienced sleeping problems, compared with 14% in the supervised group.

**Conclusion:**

These results suggest an impact of unsupervised screen time on the prevalence of mental health problems among students. Appropriate screen usage may be a major intervention target to improve children's mental health and wellbeing.

